# Physicians’ and nurses’ experience of using the Abbey Pain Scale (APS) in people with advanced cancer: a qualitative content analysis

**DOI:** 10.1186/s12912-023-01227-7

**Published:** 2023-04-04

**Authors:** Sussi Tegenborg, Per Fransson, Lisa Martinsson

**Affiliations:** 1 Department of Radiation Sciences, Oncology, Umeå University, Umeå, Sweden; 2 Department of Nursing, Umeå University, Umeå, Sweden

**Keywords:** Pain assessment, Cancer, Palliative care, End-of-life care, Abbey Pain Scale, Qualitative content analysis

## Abstract

**Background:**

The Abbey Pain Scale (APS), an observational scale used to assess pain in people with end-stage dementia, is also widely used in Sweden to assess pain in patients with advanced cancer. It is unclear whether the APS is appropriate in this context. This study aims to explore physicians’ and nurses’ experiences of using a Swedish translation of the APS (the APS-SE) in people with advanced cancer.

**Methods:**

Conventional qualitative content analysis was used to analyse interviews with physicians (n = 6) and nurses (n = 6) working in oncology and specialised palliative care about their experiences of using the APS-SE.

**Results:**

Three categories were created: fills a need, not always on target, and does not fully suit the clinical situation. Participants reported that although the APS-SE provides support in a challenging situation, it sometimes misses the mark: it does not distinguish well between pain and other types of suffering and its pain score tends not to reflect professionals’ intuitive perceptions of patients’ suffering. Some parts of the APS-SE were not considered useful, and others were perceived as ethically questionable.

**Conclusion:**

Health professionals greatly need an observational pain assessment tool for people with advanced cancer. The APS-SE is helpful in this context, but participants did not perceive it as ideal. Its problems seem inherent to the original APS rather than related to its translation from English to Swedish. Further research is needed to provide a more suitable pain assessment tool for patients with advanced cancer.

**Supplementary Information:**

The online version contains supplementary material available at 10.1186/s12912-023-01227-7.

## Background

Pain is a symptom feared and suffered by approximately 58–74% of all patients with advanced, metastatic, or terminal cancer [1–3]. Self-reported scales are considered the most reliable means of pain assessment [4, 5], but as the trajectory of cancer proceeds, many patients find it increasingly difficult to express their pain due to sedation, delirium, or imminent death [6]. Many observational scales exist for patients who can no longer self-report [7, 8], but none have been specifically developed for or evaluated for patients with cancer [3, 9].

The Abbey Pain Scale (APS) [10] is an observational assessment tool recommended by the British Geriatrics Society [11], the Australian Pain Society [12], and the Swedish National Clinical Practice Guideline for Palliative Care [13]. The APS was created in Australia in 2004 to assess pain in people with end- or late-stage dementia in residential aged care homes [10], and it is the most widely used pain assessment tool for this purpose in Australia [14]. It consists of six items: vocalisation, facial expression, change in body language, behavioural change, physiological change, and physical change. Each item offers different examples such as ‘vocalisation, e.g., whimpering, groaning, crying’. The staff assess a patient’s pain by adding the item scores for a total pain score. For example, 0–2 indicates no pain and 14–18 is considered severe pain. They complete the rating by classifying the pain as chronic, acute, or acute on chronic. The original study showed a significant correlation between the APS and nurses’ holistic pain assessments, with modest inter-rater reliability and Cronbach’s alpha of 0.74 [10].

The APS has been translated and the tested for validity in Italy [15], Japan [16], Spain [17], and Denmark [18]. Nursing home staff perceived the APS to improve systematic pain assessment [19], and Manias and colleagues suggested it could confirm self-reported pain in older patients [20]. The tool has been available in Sweden since 2011, distributed mainly through the Swedish Register of Palliative Care (SRPC) [21]. The SRPC is a national quality register that collects data about end-of-life care, especially in the last week in life, from all types of healthcare facilities in Sweden including hospitals, nursing homes, and specialised palliative care units [22]. Although there is no available literature on the subject, we know from conversation with other health care workers in palliative medicine, and from our own clinical experience, that the APS is used to assess pain in patients with diagnoses other than dementia, such as cancer in Sweden. It is often used during end-of-life care in nursing homes, in specialised palliative care units (stand-alone palliative care units, palliative hospital wards, and outpatient home care), and to some extent in hospitals. Sweden is divided into 21 different regions, each of which is responsible for healthcare in their geographic area. The SRPC has so far distributed the APS to almost 650 different health care facilities in all 21 different regions [Maria Andersson, register manager of the SRPC; personal communication].

The APS has previously been translated and culturally standardised for use in Sweden, the Abbey Pain Scale-SE (APS-SE) [23]. Other than a single case report describing the use of the APS with one cancer patient [24], we found no previous studies on the use of APS in patients with advanced cancer. This study aimed to explore physicians’ and nurses’ experience of using the APS-SE in people with advanced cancer.

## Methods

### Design and setting

Physicians and nurses (staff) were interviewed about their experiences of using the APS-SE. Purposeful sampling was used to achieve variation in occupation (physician or nurse), gender, age, workplace (stand-alone specialised palliative care unit, specialised palliative home care unit, or inpatient oncology hospital ward), and prior experience of using the APS (any Swedish version) to assess pain in people with advanced cancer. The participants were recruited by snowball sampling, approached face-to-face, and all accepted the invitation to participate. To reflect the reality of the context in which the APS is normally used, staff with a range of experience in both use of the APS and years of working with people with advanced cancer were included. Participants with little or no experience using the APS-SE were asked to use it at least twice before the interview.

All interviews were conducted in the participants’ own workplace. Prior to the start of the interview, the researcher(s) presented the reason for conducting the study and oral and written consent from the participants were collected. The interviews continued until no new essential information emerged during three consecutive interviews.

### Participants

Equal number of nurses and physicians were interviewed, of whom the majority worked within specialised palliative care. Two of the participants had less than one year’s experience working with people with cancer and the rest had more than 6 years (see Table 1).

**Table 1:** Participant demographics, n = 12

Occupation, n (%)	Physician	6 (50)
	Nurse	6 (50)
Gender, n (%)	Female	7 (58)
	Male	5 (42)
Age, range (years)		29–63
Level of education, n (%)	University studies = 2 years	2 (17)
	University studies ≥ 3 years	10 (83)
Workplace, n (%)	Oncology hospital wards	4 (33)
	Specialised palliative care units	8 (66)
Work experience with people with cancer, range (years)		0.5–31
Prior clinical experience with the APS (any Swedish version), n (%)	Yes	8 (66)
	No	4 (33)

### Interviews and analysis

A total of 12 interviews were conducted, lasting between 21 and 45 min. All the interviews were performed in the participants’ native tongue, Swedish, using a semi-structured interview guide (see Supplementary material). A pilot interview had prior been conducted to test the interview guide. At the beginning of the interview, all participants were asked to visualise a patient with advanced cancer whom they had cared for and to think of this patient while completing the APS-SE. We did not specify that the patient must also have a cognitive impairment equivalent to end-stage dementia, and we allowed the participant to reflect upon a patient of their own choice. All participants worked in care contexts attending people with advanced cancer during their last weeks or days of life.

All interviews were audio-recorded, transcribed verbatim, and analysed using conventional qualitative content analysis [25]. In one interview, an additional contact was made to clarify and extend some of the answers but contact with the other participants for clarification was not considered necessary during the analysis process. Two authors (ST, LM) began by reading the interviews to summarise their first impressions of the content. They next identified meaning units (excerpts answering the aim), which they then condensed and coded. The same authors merged all similar codes into subcategories, and all three authors (ST, PF, LM) sorted similar subcategories into three categories (see Table 2).

**Table 2:** Example of the analysis process

*Meaning unit*	*Condensed* *meaning unit*	*Code*	*Subcategory*	*Category*
I have certainly considered that, in each case, it could help an inexperienced person who has not had charge of a terminal patient before.	Help to an inexperienced person in charge of a terminal patient.	helping inexperienced	A reminder	Fills a need

We performed the analysis using QSR International’s NVivo 12 software.

### Ethical considerations

To preserve anonymity, the interviews were conducted without anyone else present besides the participants and researcher(s), and confidentiality regarding the information and the audio files was observed. The participants were also reminded that they were free to withdraw at any moment during the study.

## Results

Three categories were created during the analysis, each derived from a different set of subcategories (see Table 3).

**Table 3:** Categories and subcategories

*Subcategory*	*Category*
Supportive in a challenging situation	**Fills a need**
A reminder	
Approximate	**Not always on target**
Misleading low scores	
Pain or severe disease?	
Pain or another suffering?	
Parts less usable	**Does not fully suit the clinical situation**
Parts ethically questionable	
Disturbs relationships	

### Fills a need

The category ‘Fills a need’ were created from the subcategories ‘Supportive in a challenging situation’ and ‘A reminder’.

#### Supportive in a challenging situation

Staff members reported having clinically difficulty assessing pain in an end-of-life setting, and they saw the APS-SE as a complement to the staff’s own intuitive evaluation of the patient’s intensity of pain. It helped meet their need for more information to make an accurate pain assessment. Although participants reported using the APS-SE mainly in patients at the end of life, they also used it in patients with communicative difficulties or when they noted a persistent disparity between the patient’s self-reported pain on the Numerical Rate Scale (NRS) and their own perception of the patient’s pain. Communicative difficulties could be due to cognitive impairment, severe fatigue, or unconsciousness, and staff generally attributed discrepancies between the NRS and their own perceptions to a patient’s mild cognitive impairment or sometimes to an addiction. When the NRS results were consistently higher or lower than expected, staff used the APS-SE as a rough guide or general reference to help interpret the patient’s self-reported pain level and judge whether it seemed reasonable.

Some participants expressed that the APS-SE helped to fill their own emotional need for support. Just knowing the APS-SE was available provided them with some comfort: they literally had a piece of paper to hang onto in a difficult, emotionally demanding situation:

*If I’m feeling uncertain, like I don’t really know, then it can be a comfort to me.*

*…so that you actually have some sort of metric, sort of in the same way that it is great to have [a scale to standardise the assessment and response to acute illness], so that you can somehow still be able to assess whether what you’ve done has had any effect.*

#### A reminder

Both experienced and less experienced staff appreciated the structure provided by the APS-SE. When used as a checklist it showed less experienced staff what to pay attention to and reminded more experienced staff what not to forget:

…*when you get these suggestions, you can still get a little wake-up call.*

*I think it’s great that we’re using something like this, so that you don’t just become aware when the patient says they are in pain [...] or have the mindset that ‘well, now four hours have passed, so now they should start to feel pain’…but rather we learn to observe.*

Some of the APS-SE’s suggested examples of pain behaviours such as ‘rocking’ or ‘fidgeting’, however, were seldom considered applicable to these patients. Although the APS-SE says ‘e.g.’ before such examples, staff did not replace unsuitable examples with ones more fitting. Some participants reported that ‘checking the mouth’ and ‘examining the patient’s breathing’ were important parts of assessing these patients clinically, but these examples were not applied regularly during APS-SE assessments:

*…[our patients] seldom if ever have contractures.*

### Not always on target

The category ‘Not always on target’ were created from the subcategories ‘Approximate’, ‘Misleading low scores’, ‘Pain or severe disease?’ and ‘Pain or another suffering?’.

#### Approximate

Many participants described the final APS-SE pain score as indefinite or imprecise. Both experienced and less experienced staff members contemplated the difficulty of some of the ratings; for example, what is mild, moderate, or severe frowning? Less experienced staff worried that their ratings might be inaccurate and wondered if they would rate items differently if they had more experience. Some participants described having the final pain score expressed numerically as problematic since it gave the impression of being more exact than it was:

*But at the same time, I may not always feel that I’ve gotten it right, just because I get a total. It’s not necessarily true.*

*I may have some sort of preconception that more experienced oncologists who have been involved in numerous serious situations might possibly make a different interpretation.*

#### Misleading low scores

Participants noticed a disparity between their intuitive perceptions of patients’ suffering and the APS-SE pain scores. They often felt that the APS-SE score was too low, and when deciding between two ratings, some consistently chose the higher one. Even so, they still both felt and feared that the APS-SE assessment did not reveal the patient’s true level of pain:

*I may find that a patient is in a great deal of pain, but when I tally up the points it turns out not to be so much. I get up to moderate pain at the most, even though I think that the patient appears to be in much more pain.*

*...I tend to add a point if I’m uncertain which area I’m in. I always take the higher number just to be on the safe side.*

In a situation where the participants felt that the APS-score was too low compared to their intuitive perceptions of the patients’ pain, medications to relieve the pain were usually administrated:

*If the staff member makes the assessment that the patient is in pain, then pain relief is given or offered regardless of the score.*

#### Pain or severe disease?

Physiological changes such as pallor, sweating, or elevated temperature or pulse were often seen as signs of the severe disease itself. It was not clear to participants how to score or interpret these changes: should they rate them even if they considered them unrelated to the patient’s pain? For example, the item ‘change in body language’ often seemed to generate too few points as many patients with advanced cancer, suffering from fatigue and confined to bed, had little body language to rate. Conversely, ‘behavioural changes’ could often score too many points as clinical signs such as ‘confusion’ and ‘refusal to eat’ could generate inappropriate pain scores.

Even though the scale aimed to help the staff members make an objective assessment of the patient’s pain intensity, the participants felt that the proposed items did not always help the assessment since some of these observations were primarily interpreted as disease-related rather than signs of pain:

*...you get an altered behaviour pattern... when the patient starts to die.*

*…after all, refusing to eat or a decrease in physical activity are consequences of the situation in some way.*

Changes in body language and behaviour are often subtle. Without prior knowledge of the patient, this information must sometimes be collected from family members to ensure correct APS-SE scores:

*If it’s an end-stage cancer patient then it’s not certain that the patient will be able to move their body much at all, as their body is so weak... so this matter of rocking, fidgeting ... in this stage there are more subtle changes, I think.*

#### Pain or another suffering?

A high score on the APS-SE does indicate suffering, but many participants wondered whether it necessarily indicates pain. Since many patients with advanced cancer suffer from a ‘sludge of different symptoms’, it is not always obvious what a high APS-SE score implies. A high level of anxiety or of pain combined with anxiety or other symptoms such as dyspnoea or nausea might also yield high scores:

*The patient is troubled by something, it measures suffering… It could be a different symptom [other than pain] that is troubling the patient, such as anxiety or respiratory distress, worry.*

*...well, we don’t always know what we’re assessing. The patient may be in a late palliative phase with an extremely complex symptomatology where we think that the pain is part of the overall picture, but naturally it’s hard to know how much of it is pain...*

### Does not fully suit the clinical situation

The category ‘Does not fully suit the clinical situation’ were created from the subcategories ‘Parts less usable’ and ‘Parts ethically questionable’ and ‘Disturbs relationships’.

#### Parts less usable

The participants considered some items in the APS-SE essential, but others not particularly useful in patients with advanced cancer. All participants found the items for ‘vocalisation’ and ‘facial expression’ useful, while some simply ignored the items for ‘physiological change’ and ‘physical changes’, which they believed to have little relevance to overall assessment of pain in these patients. Some argued that the item ‘physical changes’ was mainly diagnostic and irrelevant to pain assessment. Others stated there was no reason to measure blood pressure since it added no useful information about pain in this end-of-life context:

*If I were supposed to come in and do a pain rating, I would not ask to have pulse, blood pressure and temperature measured beforehand.*

*I’m taking the blood pressure of our patients less and less often, and this pertains primarily to the cancer patients, as such measurements actually provide very little information in a late palliative stage.*

#### Parts ethically questionable

The participants emphasised the ethical importance of minimising physical examinations and procedures during the late palliative care phase to those absolutely necessary for symptom control and quality of life. Some examinations proposed by the APS-SE, such as blood pressure measurements, checking for contractures or skin problems, were seen as ethically problematic and should be avoided in the dying patient. During this phase, many patients are unconscious or too tired or cognitively impaired to decline a medical procedure or a potentially painful examination.

Thus, when the patient only had days or even hours left in life, the participants might only occasionally check a patient’s pulse or estimate their temperature while at the bedside for other reasons but did not measure blood pressure since it was considered too intrusive in this context. This understanding limited their willingness to use some parts of the APS-SE:

*…that you should test bending, stretching the joints, or turning unnecessarily. I wouldn’t want to do that.*

*... then it’s too late. We don’t do it [measure blood pressure]. When this [APS-SE] feels relevant, death has already begun... then it’s too invasive*.

*But if the patient is really, really ill, then I don’t keep on looking for bodily changes such as new pressure areas, contractures and the like, but rather I skip that, as it doesn’t seem appropriate to keep on messing about with the patient at that point.*

#### Disturbs relationships

Some participants saw the APS-SE as an obstacle in their relations with patients or with family members. In a professionally challenging situation, a strict instrument such as the APS-SE was perceived as counterproductive. It could make them appear detached and lacking in empathy towards the patient or the family members, and it could also trigger those sorts of judgements about themselves:

*If the situation feels uncertain and I need to offer comfort even if I’m not feeling it myself... if I were to then pull out an instrument and starting ticking boxes, then I would feel that I was not offering comfort.*

There were no major differences between the physicians’ and nurses’ experiences of using the APS-SE, except the physicians emphasised the importance of using the APS-SE to re-evaluate pain after a patient is given pain medication.

## Discussion

This study showed that the APS-SE gave medical staff some support and structure in a clinically and emotionally challenging situation, but some items were not considered suitable for people with advanced cancer. Some examinations were considered ethically questionable to perform in a dying patient and overall, the instrument left the feeling of not really being on point when used in this context. Thus, the APS-SE is not fully transferable from people with dementia to people with advanced cancer. We interpret this problem as inherent to the original APS [10] rather than to its scientific translation and cultural adaptation to the APS-SE. To our knowledge, this is the first study on the usefulness of the APS on this population.

In a survey by the Eastern Cooperative Oncology Group (ECOG), 76% of physicians considered inferior pain assessment the most important obstacle to proper pain management [26]. In the absence of a patient’s ability to self-report, observational pain assessment instruments such as the APS-SE become necessary. The APS was developed specifically for people with dementia, and some of the examples provided in the instrument are not suitable for people with advanced cancer, leading to lower scores in this population than expected. The APS was also reported to be difficult to use when tested in the emergency department [27], but it was found reliable for more limited conditions such as low back pain [28] or osteoarthritic pain during exercise [29].

Participants considered some parts of the APS-SE irrelevant to assessing pain. Many ignored the item ‘physiological changes’ and simply did not execute the proposed physical examination. We believe that this should not be interpreted as a problem of implementation or a lack of education or guidelines. Instead, the staff understood that such examinations are not good indicators of pain in this context. This perception was endorsed by a study of the APS’s psychometric qualities in nursing home residents with osteoarthritic pain during exercise [29], which suggested removing the item ‘physiological change’.

The participants’ understanding that physical examinations did not contribute to the pain assessment increased their feeling that such examinations were ethically questionable, especially at the end of life. Their refusal to perform such examinations, combined with the instrument’s sometimes inappropriate examples for patients with advanced cancer, could help explain the participants’ feeling that patients’ total pain scores were lower than they should be. Participants were left feeling they had missed the mark and believing that the APS-SE does not accurately reflect the patient’s pain. A study of the feasibility and clinical utility of the Japanese version of APS also showed a gap between self-reported pain and APS scores [30].

Participants felt that the APS-SE score was a better indicator of suffering in general than of pain in particular. A crucial problem, the difficulty of distinguishing between pain and anxiety, has been noted by others [19]. Not knowing whether the patient is suffering pain or something else makes it difficult to decide how to provide relief. Several items also focus on ‘changes’ that require prior knowledge of the patient. This information must sometimes be collected from family members, as described in an article on family/caregiver roles in caring for cognitively impaired older people in pain [31]. Since it is imperative to have a functioning relationship with the patient’s family, some of the participants expressed concern that the use of a ‘checklist’ would disturb the relationship between the staff and the patient and/or the family members. While it cannot be stated in this study whether this view can be generalised to more instruments and clinical contexts, the context-relevant expression of concern of not being sensitive while caring for a dying patient is important and should be considered when bringing instruments into palliative care.

Observational scales for pain assessment are used when patients cannot use self-reporting scales such as the Numerical Rating Scale (NRS), Visual Analogue Scale (VAS), or Verbal Rating Scale (VRS). The ability to self-report pain is usually lost earlier in the disease trajectory of dementia than cancer. People with advanced cancer can still have enough cognition to use self-reporting scales late in the disease trajectory, but near the end of their lives might suffer such severe fatigue that they can no longer use the NRS, VAS, or VRS. Thus, the period in which an observational scale is useful is longer in people with dementia, who could still be in relatively good physical health when an observational scale is required for pain assessment. The participants in this study also reported that advanced cancer can itself yield erroneous measurement values for individual items that can lead to total APS scores that are too high or too low. We believe that the difference in overall physical health between the different populations, combined with the risk of invalid cancer-generated measurements, mean the APS is more appropriate for people with dementia than for people with advanced cancer.

Many participants rated the APS-SE as just ‘okay’ for use in people with advanced cancer, but continue to use it, mainly because they currently have no alternative. Further studies are necessary to test the psychometric qualities of the APS-SE for assessing pain in people with advanced cancer.

### Strengths and limitations

We used an established guideline for qualitative content analysis [25]. This conventional type of qualitative content analysis is considered appropriate when pre-existing knowledge of the area of study is very limited [32]. One of the authors (LM) has previous experience in the method [33]. The number of interviews needed until nothing essentially new emerged in this study was in line with the numbers needed in prior research [34].

During the interview participants were asked to complete the APS-SE while visualising a particular patient they had cared for. The only characteristic we specified for the chosen patient was advanced cancer because we wanted to explore the staff’s experience of using the APS in this care context without restrictions. This open approach made it obvious that the APS is used not only in cancer patients with cognitive impairment, but also in patients suffering from fatigue or near death and to check whether self-reported pain levels seem reasonable. If participants had been asked only to visualise a cancer patient with severe cognitive impairment, this information would have been lost.

All participants were currently working or had previously worked clinically with at least one of the authors. Interviewing team members may raise the question of trustworthiness. Team members might be afraid of losing collegial esteem if they express themselves freely when discussing a controversial subject [35]. Since we consider the topic neither very controversial nor too private, we assess the risk of participants feeling unable to express themselves freely as low.

When clinically implemented, the pros and cons of using the instrument were probably discussed among staff members. These discussions were likely resumed in the health care units during this study. The sample size is small and from the same geographical area which could lead to bias regarding these presumed discussions. To minimise this, participants were recruited from three different workplaces.

## Conclusion

This study revealed a great need in advanced cancer care for a pain assessment tool, especially for those who are very ill and/or can no longer verbalise their pain. The APS-SE gives staff some support, but it is not perceived as ideal in this context. Its problems seem inherent to the original APS and should not be attributed to its translation from English to Swedish. Further research is necessary to provide a more suitable pain assessment tool for patients with advanced cancer.

## Electronic supplementary material

Below is the link to the electronic supplementary material.

Supplementary Material 1

## Data Availability

Data will not be available since this was not included in the ethical approval and the participants were not asked for consent to share raw data.

## Abbreviations

APS: Abbey Pain Scale
APS-SE: a Swedish version of the APS
EAPC: European Association for Palliative Care
NRS: Numerical Rating Scale
SRPC: the Swedish Register of Palliative Care
VAS: Visual Analogue Scale
VRS: Verbal Rating Scale.
