# Supplementary material for: Physicians’ and nurses’ experience of using the Abbey Pain Scale (APS) in people with advanced cancer: a qualitative content analysis
Source: BMC Nurs. 2023 Apr 4;22:95. doi: 10.1186/s12912-023-01227-7 (PMC10071650; doi:10.1186/s12912-023-01227-7)
Supplement: Supplementary file 1 — Supplementary Material 1 [file 12912_2023_1227_MOESM1_ESM.docx]

# Semi-structured interview guide:

Study number:

Profession:

Age:

Female □ Male □ Other □

Total number of years in the profession:

Number of professionally active years in oncology and/or specialist palliative care:

Prior clinical experience with the APS (any version):

May the researchers contact the subject during or after the analysis process with questions?

**Interviewer instructions**

Begin by presenting the APS and explain that it was developed in dementia care for patients who are unable to verbalise pain. If not using APS-SE in the clinic, point out the difference between the version used at the relevant clinic and the version the study aims to evaluate, the APS-SE. Explain that the purpose is to explore physicians’ and nurses’ experience of using the APS-SE in people with advanced cancer. Ask the interviewee to visualise a patient with advanced cancer whom they had cared for and then think out loud while completing the APS-SE.

Ask the interview questions below during the interview. If necessary, use follow-up questions such as ‘can you elaborate your answer’.

## Interview questions

1. Can you describe a situation in which you think the APS worked well?
2. Can you describe a situation in which you think the APS did not work well?
3. What is your experience of using the APS?
4. What is your opinion of the APS?
5. What do you think of question 1?
6. What do you think of question 2?
7. What do you think of question 3?
8. What do you think of question 4?
9. What do you think of question 5?
10. What do you think of question 6?
11. For which patients do you consider the use of the APS appropriate?
12. For which patients do you consider the use of the APS inappropriate?
13. Do you think the APS accurately measures pain for patients with advanced cancer who are unable to use the NRS? Explain your viewpoint.
14. What parts of the form do you find potentially confusing or difficult to understand?
15. What, if anything, would you phrase differently?
16. What parts of the form could potentially be perceived as objectionable or problematic by staff, patients or family members?
17. What other difficulties have you experienced with the APS, if any?
18. What aspects or dimensions of pain and its expression would you add, if any?
19. What aspects or dimensions that are not addressed in the APS might be included in your assessment of the patient?
20. What additional response options would you like to include, if any?
21. Can all of the questions always be answered? Explain your viewpoint.
22. Do you take blood pressure/pulse/temp? Why or why not?
23. When you are finished with your evaluation, how do you make your assessment? Do you use the total sum or the individual components to assess whether the patient has pain?
24. How do you communicate the results you have reached using the APS to other medical staff?

(If they do not use a number, ask: Why do you not use a number?)

1. How do you use the APS after an intervention has been performed?
2. How do you think your assessment/evaluation compares to that of another APS user?
3. How do you view any discrepancies between your and the other person’s evaluation?
4. If the patient could use the APS, how do you think your evaluation would compare to the patient’s evaluation?
5. How do you view any discrepancies between your and the patient’s evaluation?
6. In what situation would you use the APS on a patient who could use the VAS or the NRS?
7. In what situation do you not use the APS?
8. What leads you to use the APS?
9. How do you use the question about type of pain?
10. Can **you** summarize what you think is most important? Would you like to add anything?
11. Now that we have completed the interview – do we still have your consent to use the interview?
